# Rhino-Orbital Mucormycosis With Delayed Diagnosis in an Immunocompromised Patient: A Case Report

**DOI:** 10.7759/cureus.84702

**Published:** 2025-05-23

**Authors:** Lenyn Daniel Montes Sevilla, Francisco Alvarado Alvarez, Stephanie Hernández Camacho, Hassler Stefan Macias Sánchez, Alejandro Alfaro Goldaracena

**Affiliations:** 1 General Surgery, Instituto Nacional de Ciencias Médicas y Nutrición Salvador Zubirán, Mexico City, MEX; 2 Surgical Oncology, Instituto Nacional de Ciencias Médicas y Nutrición Salvador Zubirán, Mexico City, MEX; 3 Surgery, Instituto Nacional de Ciencias Médicas y Nutrición Salvador Zubirán, Mexico City, MEX; 4 Medical Education, Instituto Nacional de Ciencias Médicas y Nutrición Salvador Zubirán, Mexico City, MEX

**Keywords:** case report, covid-19, diabetes mellitus, immunocompromised host, mucormycosis

## Abstract

Rhinoorbital mucormycosis is a rapidly progressing invasive fungal infection associated with high mortality, primarily affecting immunocompromised patients. We present the case of a 78-year-old male patient with a history of type 2 diabetes mellitus, systemic arterial hypertension, and kidney transplantation, who was admitted for severe pneumonia due to SARS-CoV-2 at the National Institute of Medical Sciences and Nutrition "Salvador Zubirán," a care center in Mexico City. During his hospital stay, he developed a subacute left orbital lesion. The diagnosis of mucormycosis was established based on histopathological findings compatible with mucormycosis, without microbiological or molecular confirmation. Treatment consisted of liposomal amphotericin B and staged surgical debridements, avoiding orbital exenteration. Despite the complex clinical course and the absence of culture or PCR confirmation, the patient experienced satisfactory functional recovery, preserving visual acuity and without relevant aesthetic sequelae. This case, managed at a national referral hospital in Mexico, emphasizes the importance of considering mucormycosis in the differential diagnosis of orbital lesions in patients with multiple risk factors and highlights the value of a timely, multidisciplinary approach, particularly in settings with limited diagnostic resources.

## Introduction

Mucormycosis is a fulminant opportunistic fungal infection with an overall prevalence ranging between 0.005 and 1.7 per million inhabitants [1]. It is caused by filamentous fungi of the order *Mucorales*, predominantly of the genera *Rhizopus*, *Mucor*, and *Lichtheimia*, classified within the phylum *Mucoromycota* [2]. These fungi are present in the environment and generally do not cause serious problems in humans but can lead to severe infections in immunocompromised individuals [3,4]. The clinical manifestations of mucormycosis are varied and include sinusitis (such as pansinusitis, rhinoorbital, or rhinocerebral disease), as well as skin, gastrointestinal, pulmonary, and disseminated infections [3,5,6]. This mycosis is characterized by its rapid progression, angioinvasive capacity, and high mortality rate [7].

Immunocompromised patients, including those with poorly controlled diabetes mellitus, metabolic acidosis, use of corticosteroids or immunosuppressants, and solid organ transplant recipients, constitute the highest risk group [8]. During the COVID-19 pandemic, a significant increase in the incidence of mucormycosis was observed, attributed to the immunomodulatory effect of SARS-CoV-2, diabetic decompensation, widespread use of steroids, and disruption of epithelial barriers [9,10].

Mucormycosis in its rhino-orbital form initially affects the paranasal sinuses, with secondary extension to the eye socket, and can progress to the central nervous system, where it is classified as rhino-orbito-cerebral mucormycosis. Its clinical presentation usually includes facial pain, periorbital edema, proptosis, chemosis, palatal ulceration, cranial nerve palsy, or loss of vision, requiring a high degree of clinical suspicion for timely diagnosis [11,12].

Rhinoorbital mucormycosis represents the most common clinical form of COVID-19-associated mucormycosis, with a prevalence ranging from 45% to 74% of cases reported during the pandemic in countries with a high disease burden, such as India and Mexico [10,12]. This entity is associated with a high mortality rate, estimated between 25% and 62%, which can exceed 80% when there is brain involvement [13].

Among the most frequent risk factors are poorly controlled diabetes mellitus (up to 80% of cases), corticosteroid use (76%), recent SARS-CoV-2 infection (65%), and immunosuppression due to kidney transplantation or cytotoxic treatments (20%-25%) [10,14].

Despite its aggressiveness, the definitive diagnosis of rhinoorbital mucormycosis is established primarily through histopathological examination with specific stains, as fungal cultures and molecular tests have limited sensitivity and may be negative in up to 40% of cases [13,15].

Treatment requires aggressive and early surgical intervention, along with the use of active antifungals such as liposomal amphotericin B, as agents like voriconazole are not effective against Mucorales [16]. Misidentification of pseudohyphae, such as those seen in Candida infections, can lead to inappropriate antifungal therapy and worsen the prognosis [15].

Among patients who survive this infection, up to 70% have functional or aesthetic sequelae, including vision loss (40%) and orbitofacial deformities that may require reconstruction [10,14]. These figures underscore the importance of early diagnosis and aggressive antifungal and surgical treatment, supported by a multidisciplinary approach.

In this study, we present the case of an immunocompromised patient with a history of kidney transplantation, diabetes mellitus, and COVID-19 infection, who developed chronic rhinoorbital mucormycosis with an atypical clinical presentation and inconclusive histopathological findings in the initial stages. This case highlights the diagnostic challenges, the importance of active surveillance in patients with multiple risk factors, and the consequences of inappropriate antifungal treatments.

## Case presentation

We present the case of a 78-year-old Mexican male patient with a personal history of type 2 diabetes mellitus (DM2), systemic arterial hypertension, kidney transplantation from a living donor in 2006 due to diabetic nephropathy (treated with tacrolimus 1 mg/day and prednisone 5 mg/day), and previous respiratory infections (including community-acquired pneumonia). The patient had a complete vaccination schedule for childhood immunizations and COVID-19, as well as vaccinations against pneumococcus and influenza. On admission, his respiratory rate was 36 rpm, blood pressure was 150/70 mmHg, and oxygen saturation was 85% with a reservoir mask. He exhibited intercostal retraction and use of accessory muscles. A renal graft scar was evident, with no tenderness on palpation. Eye examination revealed conjunctival edema, induration, and local hyperthermia without crepitus. The complete clinical course is summarized in Table 1.

**Table 1:** Clinical timeline of the case. Chronological sequence of key clinical events in the patient, including diagnostic milestones, therapeutic interventions (antifungal and surgical), and progression until hospital discharge.

Date	Relevant Clinical Event
09 June 2023	Admission due to severe SARS-CoV-2 pneumonia; ICU management with mechanical ventilation, remdesivir, and dexamethasone.
25 June 2023	Onset of a lesion at the medial canthus of the left eye; Ophthalmology rules out endogenous ocular infection.
30 June 2023	Progression of the lesion with inflammatory signs. Cranial CT reveals chronic left-sided pansinusitis with inflammatory changes in extraconal fat and orbital soft tissues, along with microangiopathy.
03 July 2023	First paranasal sinus biopsy shows findings suggestive of mucormycosis.
09 July 2023	Additional biopsy without molecular confirmation; clinico-pathological diagnosis confirmed in a multidisciplinary session.
14 July 2023	Initiation of liposomal amphotericin B and surgical resection of necrotic orbitonasal tissue.
29 August 2023	Isolation of E. faecalis; antibiotic treatment initiated.
11 September 2023	Negative margins for mucormycosis. Antifungal therapy discontinued.
12 October 2023	Patient discharged after more than 100 days of hospitalization. Favorable evolution without visual loss or significant functional or aesthetic sequelae.

**Figure 1:**
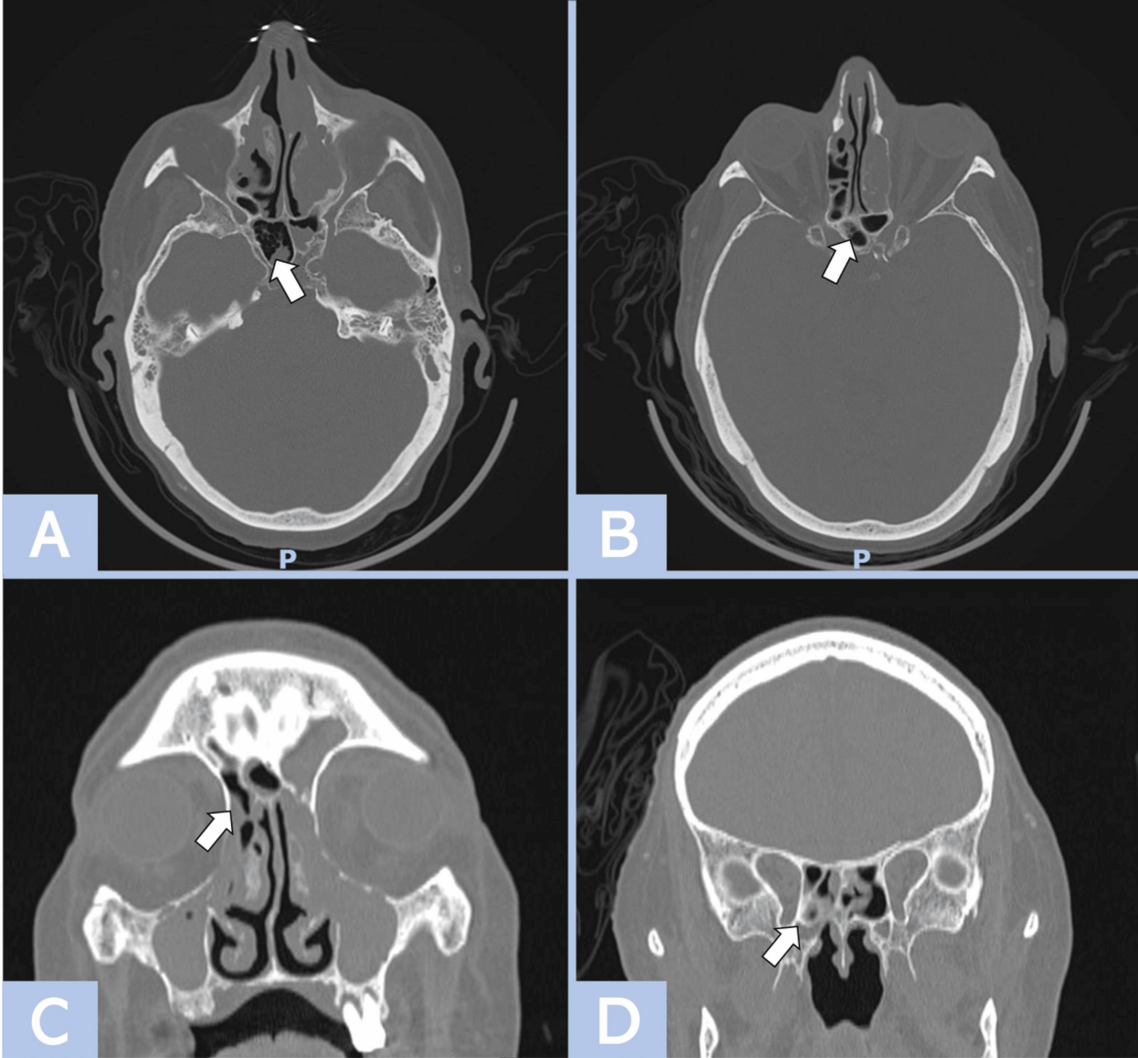
Plain skull CT revealing inflammatory changes. Plain skull CT, performed with axial (A-B) and coronal (C-D) cuts and viewed through a bone window, shows opacification of the maxillary, ethmoid, sphenoid, and frontal sinuses. The presence of air bubbles (marked with arrows) within the opacified areas suggests an acute process. Evidence of osteitis is observed in the ethmoid cells. Inflammatory changes are noted in the left orbit, including striation of the extraconal fat and eyelid edema, leading to proptosis. No fluid collections are identified. Extraocular muscles appear to be of similar thickness compared to the contralateral side.

He was transferred to the National Institute of Medical Sciences and Nutrition "Salvador Zubirán" for severe pneumonia associated with SARS-CoV-2, confirmed by RT-PCR and CT scan. He was admitted to the ICU due to hypoxemia, requiring orotracheal intubation, prone positioning, and treatment with remdesivir, dexamethasone, and antibiotics (meropenem, piperacillin-tazobactam, and vancomycin). After 16 days of hospitalization, a lesion was identified in the region of the internal canthus and middle third of the lower eyelid of the left eye, characterized by a haematic crust, edematous conjunctiva of the ipsilateral eye, and induration with slight hyperthermia but no crepitus on palpation. The bulbar conjunctiva exhibited inferior and temporal chemosis. The Ophthalmology Department ruled out endogenous ocular infection. Subsequently, the lesion progressed with orbital inflammatory signs that prompted a cranial tomography, which showed chronic pansinusitis with left-side predominance, associated with inflammatory changes in the extraconal fat and soft tissues of the ipsilateral orbital region with microangiopathy (Figure 1).

The patient was then evaluated by the Dermatology service, which requested a biopsy of the lesion due to suspicion of soft tissue infection. He was taken to the operating room, where a biopsy of the maxillary sinus and orbital tissue was obtained. Histopathological analysis of the tissue revealed broad, non-septate hyphae with right-angle branching-features characteristic of fungi belonging to the order Mucorales. Periodic acid-Schiff (PAS) staining highlighted hyphae measuring 5 to 10 microns in diameter with thin walls, while Grocott-Gomori methenamine silver (GMS) staining demonstrated the presence of hyphae in close association with muscle fibers, suggesting a pattern of angioinvasion and active tissue infiltration. These findings confirmed the presence of mucormycosis in the biopsy specimen.

A new biopsy of the lesion at the inner canthus and lower left eyelid was requested. Amplification of genetic material and sequencing of both biopsies were performed to detect Mucorales; however, no result was obtained due to failed amplification. The case was thoroughly evaluated in a multidisciplinary team session, which concluded that the diagnosis of mucormycosis was proven based on histological evidence.

Surgical treatment and debridement of the affected tissue were planned. The patient received antifungal treatment with liposomal amphotericin B. He underwent surgery with resection of necrotic tissue from the eyelids and internal canthus, as well as necrotic nasal and maxillary bone mucosa. Multiple surgical washes and new biopsies were performed. Subsequently, ampicillin-sensitive Enterococcus faecalis was identified, prompting adjustment of antimicrobial therapy to amoxicillin with clavulanic acid. Given the negative margins for mucormycosis, amphotericin B therapy was discontinued.

**Figure 2:**
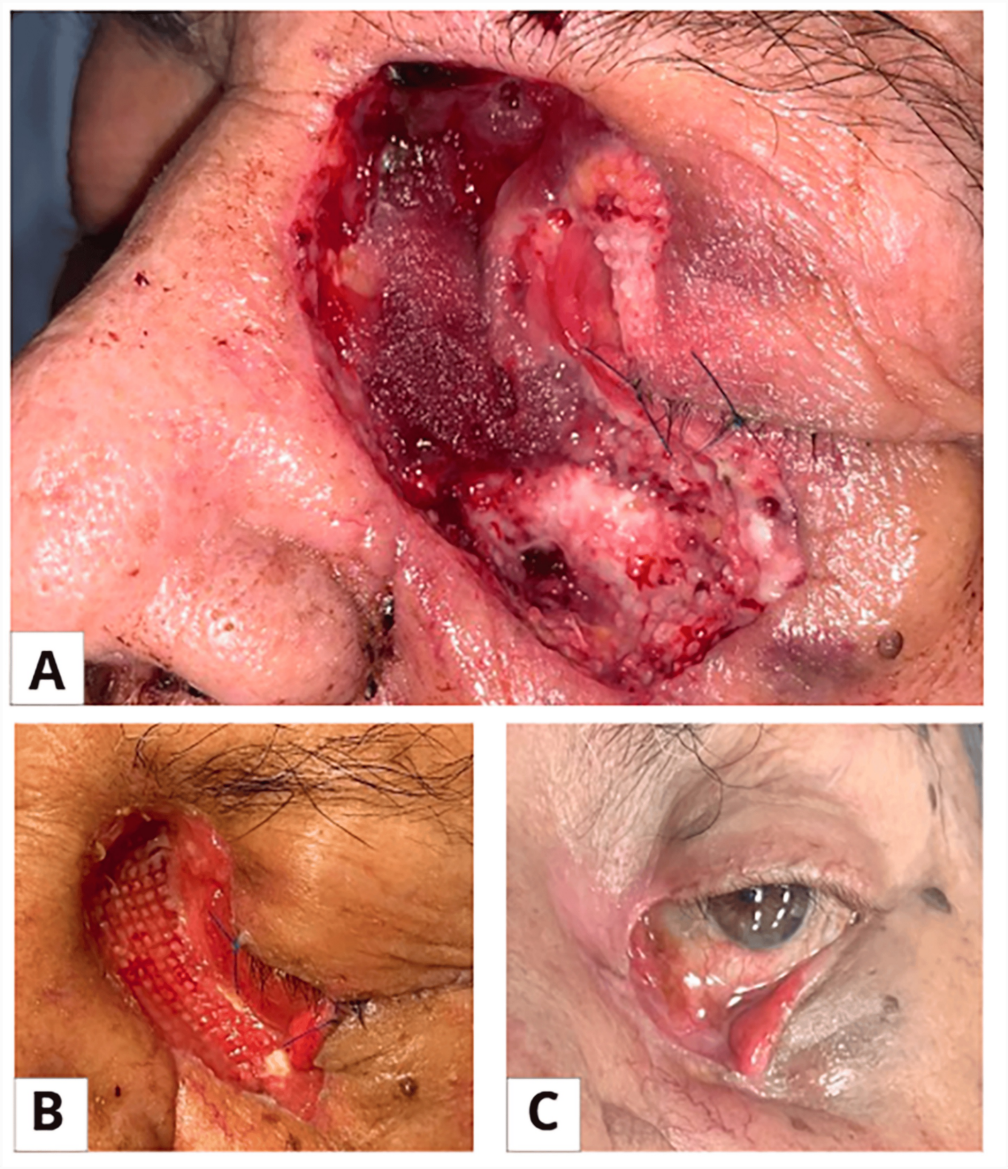
Clinical evolution of the rhino-orbital lesion following surgical and antifungal treatment. A) Preoperative image: lesion at the medial canthus and lower left eyelid with edema, hematic crust, and induration, suggestive of rhino-orbital mucormycosis. B) Immediate postoperative image following surgical debridement of necrotic orbitonasal tissue and initiation of liposomal amphotericin B. C) Image at hospital discharge (>100 days): favorable evolution without loss of visual acuity, with appropriate healing and no significant aesthetic sequelae.

After more than 100 days of hospitalization, the patient showed favorable clinical progress. He did not require orbital exenteration, maintained visual acuity, and had no major functional or aesthetic sequelae (Figure 2). He was discharged with outpatient follow-up.

Antifungal treatment was well tolerated by the patient, with no severe adverse events related to the administration of liposomal amphotericin B. Adherence was ensured through continuous hospital surveillance, and no significant interruptions in therapy were documented. Regular renal and liver function tests were conducted to monitor for toxicity, with a stable clinical course observed.

The patient described the process as “exhausting and painful,” but expressed gratitude to the medical team for their joint efforts, which allowed him to preserve his vision. He appreciated the consistent communication with his family and the empathetic care he received throughout his extended hospitalization. He expressed a desire to share his experience in the hope that it may help other patients receive timely diagnosis and treatment.

## Discussion

Rhino-orbital mucormycosis is an opportunistic infectious entity with fulminant progression, caused by fungi of the order *Mucorales*, previously classified within the Zygomycetes. Among the most relevant pathogenic genera are *Rhizopus*, *Mucor*, and *Lichtheimia*, which exhibit a particular tropism for tissues with hypoxia, acidosis, and increased serum iron availability [2]. Rhino-orbital mucormycosis has gained clinical prominence since the onset of the COVID-19 pandemic due to the exponential rise in its incidence, particularly in patients with metabolic comorbidities, corticosteroid exposure, and iatrogenic immunosuppression [9,10,14].

It has been proposed that impairment of innate defense mechanisms, such as ciliary clearance and T cell function, plays a crucial role in the pathophysiology of mucormycosis, especially in patients with COVID-19-related acute respiratory distress syndrome [17]. Likewise, neutrophils, as the first line of defense against filamentous fungi, may exhibit dysfunction in this context, which, when combined with hyperglycemia and acidosis, facilitates fungal invasion [18]. Even short courses of corticosteroids have been implicated as a precipitating factor in susceptible individuals [19]. SARS-CoV-2-induced immunosuppression, prolonged corticosteroid use, and metabolic stress-related hyperglycemia contribute collectively to the increased risk of rhino-orbital mucormycosis [20,21].

Numerous studies have documented a direct correlation between the pandemic and the rise in this mycosis. Patel A et al. reported a doubling of mucormycosis cases in India during the pandemic period, while Chang CY et al. described the first documented case in Malaysia, evidencing the geographic expansion of this infection in clinical contexts associated with hyperglycemia and immunosuppression [9,22]. In Latin America, including Mexico and Brazil, prevalence has also increased, with a compatible clinical profile observed in patients with type 2 diabetes mellitus, chronic kidney disease, kidney transplantation, and recent SARS-CoV-2 infection [23].

The case presented here involves a patient with multiple recognized risk factors: type 2 diabetes mellitus, chronic kidney disease secondary to kidney transplantation, and pharmacological immunosuppression, in addition to COVID-19 infection. The pathophysiology of rhino-orbital mucormycosis in this context involves multiple mechanisms: persistent hyperglycemia, metabolic acidosis, neutrophil dysfunction, increased circulating free iron, and local epithelial damage, all of which facilitate tissue invasion by *Mucorales* [11,12,24]. The heightened susceptibility in these patients results from both disruption of innate barriers and an impaired adaptive immune response.

A notable feature in this case was the subacute form of presentation, without palatal necrosis or initial visual loss. Similar cases have been described in the literature, often misdiagnosed initially as orbital cellulitis or bacterial sinusitis [13]. Early identification is crucial, but the nonspecific nature of symptoms can complicate timely diagnosis [25].

From the diagnostic point of view, the histopathological finding of broad, sparsely septate hyphae with irregular branching is considered the reference criterion for the diagnosis of mucormycosis. However, in the early stages of the disease, nonspecific inflammatory changes, extensive avascular necrosis, or fragmentation of fungal structures can make interpretation difficult, especially in the absence of special stains such as PAS or GMS [15,26].

After the confirmation of fungal hyphae, cultures, immunotransferable molecular tests, and/or histopathological studies of the tissue are necessary to specify the microorganism [27]. In this regard, imaging studies play a fundamental role in assessing the extent of the disease, but the definitive diagnosis is usually based on histopathological examination of biopsies [28]. In our patient, both cultures and molecular tests were requested; however, the necessary material for these tests was not available. This reinforces the importance of histopathology as a primary diagnostic tool, especially when clinical and radiological findings are suggestive.

Although the diagnosis of mucormycosis was supported by conclusive histopathological findings, including the observation of broad, non-septate hyphae with right-angle branching visualized through PAS and GMS staining, it was not possible to include representative microscopic images in this report due to the lack of institutional consent for the dissemination of histological material from the clinical archive. Despite this limitation, the official pathology report issued by the Department of Pathology at the Instituto Nacional de Ciencias Médicas y Nutrición “Salvador Zubirán” was incorporated into the patient’s clinical file and reviewed during a multidisciplinary meeting to guide therapeutic decisions.

Timely initiation of antifungal therapy is essential. Liposomal amphotericin B remains the mainstay of treatment, while newer triazoles such as posaconazole and isavuconazole have emerged as potential alternatives [29,30]. Surgical debridement of necrotic tissue also remains a cornerstone of treatment, aiming to limit fungal invasion and improve antifungal penetration. However, the extent of surgery must be balanced against the risk of functional and aesthetic morbidity [31].

A particularly noteworthy clinical aspect of this case was the preservation of visual acuity and anatomical viability of the eyeball, despite the extent of the infection in the orbital region. In published series, the rate of orbital exenteration in rhino-orbital mucormycosis can reach 40-50%, especially in patients with delayed diagnosis, cavernous sinus involvement, or intracranial progression [12,32]. In this context, the favorable outcome observed reflects the effectiveness of a timely, conservative yet complete surgical approach, complemented by appropriate and prolonged antifungal therapy.

The decision to avoid orbital exenteration was made carefully in a multidisciplinary setting, supported by clinical, radiological, and surgical findings that suggested containment of the infection without intraocular involvement. This approach preserved visual function without compromising infection control, representing an optimal balance between therapeutic radicalism and functional preservation. Comparatively, this outcome represents a positive exception within the typical clinical spectrum of rhino-orbital mucormycosis in severely immunocompromised patients [33,34].

The therapeutic success in this case was due to the implementation of a multidisciplinary strategy combining systemic antifungal treatment (liposomal amphotericin B and isavuconazole) with stepwise surgical debridement. This approach, widely supported by international guidelines, led to clinical eradication without ocular functional impairment, an outcome that is uncommon in severely immunosuppressed patients [12,16].

Unlike other previously documented case reports, this case presents several distinctive elements: an insidious and atypical clinical presentation, complete absence of microbiological confirmation, diagnosis sustained solely by histopathological findings, preservation of visual acuity despite extensive infection, and complete resolution in a referral center in Latin America. These characteristics make this report a valuable contribution to the literature on rhino-orbital mucormycosis, especially in settings with limited diagnostic resources, and reinforce the importance of maintaining a high index of clinical suspicion in patients with multiple risk factors.

## Conclusions

This case highlights the importance of a multidisciplinary approach for the timely diagnosis and management of rhino-orbital mucormycosis in immunocompromised patients. One of the main strengths of this report lies in the comprehensive, team-based strategy employed to manage a severe condition, resulting in the preservation of visual acuity and systemic stability in a high-risk patient. However, one of the limitations was the absence of histopathological imaging, as well as the lack of microbiological confirmation through culture or molecular amplification of *Mucorales*, which prevented definitive identification of the causative agent. Despite this, the diagnosis was strongly supported by the official histopathological report.

There is a clear need to improve access to specialized microbiological testing for the diagnosis of mucormycosis across all levels of care. This case also underscores the importance of maintaining a high index of suspicion in at-risk patients and ensuring timely access to diagnostic tools. Additionally, further studies are required to develop effective prevention and treatment strategies tailored to this vulnerable patient population.
